# Supplementary material for: A decision point between transdifferentiation and programmed cell death priming controls KRAS-dependent pancreatic cancer development
Source: Nat Commun. 2025 Feb 19;16:1765. doi: 10.1038/s41467-025-56493-7 (PMC11839950; doi:10.1038/s41467-025-56493-7)
Supplement: Supplementary file 1 — Supplementary Information [file 41467_2025_56493_MOESM1_ESM.pdf]

Supplementary Information

A decision point between transdifferentiation and programmed cell death priming controls KRAS-dependent pancreatic cancer development

Anne T. Schneider[1], Christiane Koppe[1], Emilie Crouchet[2], Aristeidis Papargyriou[3,4,5], Michael T. Singer[1], Veronika Büttner[1], Leonie Keysberg[1], Marta Szydlowska[6], Frank Jühling[2], Julien Moehlin[2], Min-Chun Chen[4], Valentina Leone[3,6,7], Sebastian Mueller[8,9], Thorsten Neuß[10], Mirco Castoldi[1], Marina Lesina[11], Frank Bergmann[12,13], Thilo Hackert[14,15], Katja Steiger[16], Wolfram T. Knoefel[17], Alex Zaufel[1], Jakob N. Kather[18,19,20], Irene Esposito[21], Matthias M. Gaida[22,23,24,25], Ahmed Ghallab[26,27], Jan G. Hengstler[26], Henrik Einwächter[4], Kristian Unger[28,29], Hana Algül[11], Nikolaus Gassler[30], Roland M. Schmid[4], Roland Rad[9,31], Thomas F. Baumert[2,32,33,34], Maximilian Reichert[3,4,35,36,37], Mathias Heikenwalder[6,38], Vangelis Kondylis[1]*, Mihael Vucur[1]* and Tom Luedde[1,39]*§

[1]Department of Gastroenterology, Hepatology and Infectious Diseases, University Hospital Düsseldorf, Medical Faculty at Heinrich-Heine-University, Duesseldorf, Germany; [2]University of Strasbourg, Inserm, Institute for Translational Medicine and Liver Disease (ITM), UMR_S1110, Strasbourg, France; [3]Translational Pancreatic Cancer Research Center, Klinik und Poliklinik für Innere Medizin II, Klinikum rechts der Isar, Technical University of Munich, Munich, Germany; [4]Klinik und Poliklinik für Innere Medizin II, Klinikum rechts der Isar, Technical University of Munich, Munich, Germany; [5]Institute of Stem Cell Research, Helmholtz Center Munich, German Research Center for Environmental Health, Neuherberg, Germany; [6]Division of Chronic Inflammation and Cancer, German Cancer Research Center (DKFZ), Heidelberg, Germany; [7]Research Unit Radiation Cytogenetics, Helmholtz-Zentrum München, German Research Center for Environmental Health, Neuherberg, Germany; [8]Institute of Molecular Oncology and Functional Genomics, School of Medicine, TU Munich, Munich, Germany. [9]Center for Translational Cancer Research (TranslaTUM), School of Medicine, Technical University of Munich, Munich, Germany; [10]Lehrstuhl für Biophysik E27, Center for Protein Assemblies (CPA), Technical University Munich (TUM), Garching, Germany; [11]Comprehensive Cancer Center München, Chair for Tumor Metabolism, TUM University Hospital, Klinikum rechts der Isar, Technical University of Munich, School of Medicine and Health, Munich, Bavaria, Germany; [12]Institut of Pathology, Heidelberg University Hospital, Germany; [13]Department of General, Visceral, and Transplantation Surgery, University Hospital Heidelberg, Heidelberg, Germany; [14]Clinical Pathology, Klinikum Darmstadt GmbH, Darmstadt, 64283, Germany; [15]Department of General, Visceral and Thoracic Surgery, University Hospital Hamburg-Eppendorf, Hamburg, Germany; [16]Institute of Pathology, School of Medicine, Technical University of Munich, Munich, Germany; [17]Department of Surgery A, Heinrich-Heine-University Düsseldorf and University Hospital Düsseldorf, Duesseldorf, Germany; [18]Else Kroener Fresenius Center for Digital Health (EFFZ), Technical University Dresden, Dresden, Germany; [19]Division of Pathology and Data Analytics, Leeds Institute of Medical Research at St James's, University of Leeds, Leeds, UK; [20]Medical Oncology, National Center for Tumor Diseases (NCT), University Hospital Heidelberg, Heidelberg; [21]Institute of Pathology, University Hospital Duesseldorf, Heinrich-Heine University, Duesseldorf, Germany; [22]Institute of Pathology, University Medical Center Mainz, JGU-Mainz, Mainz, Germany; [23]Research Center for Immunotherapy, University Medical Center Mainz, JGU-Mainz, Mainz, Germany; [24]Joint Unit Immunopathology, Institute of Pathology, University Medical Center, JGU-Mainz; [25]TRON, Translational Oncology at the University Medical Center, JGU-Mainz, Mainz, Germany; [26]Leibniz Research Centre for Working Environment and Human Factors (IfADo) at the Technical University Dortmund, Germany; [27]Forensic Medicine and Toxicology Department, Faculty of Veterinary Medicine, South Valley University, Qena, Egypt; [28]Department of Radiation Oncology, University Hospital, LMU Munich, Munich, Germany; [29]Research Unit Translational Metabolic Oncology, Institute for Diabetes and Cancer, Helmholtz Zentrum München Deutsches Forschungszentrum für Gesundheit und Umwelt (GmbH), Neuherberg, Germany; [30]Section Pathology of the Institute of Forensic Medicine, University Hospital Jena, Jena, Germany; [31]Department of Internal Medicine II, Klinikum Rechts der Isar, Technical University of Munich, Munich, Germany; [32]Pôle des Pathologies Hépatiques et Digestives, Service d'Hepato-Gastroenterologie, Strasbourg University Hospitals, Strasbourg, France; [33]Institut Hospitalo-Universitaire (IHU) Strasbourg, France; [34]Institut Universitaire de France (IUF), Paris, France; [35]Center for Organoid Systems (COS), Technical University of Munich, Garching, Germany; [36]Munich Institute of Biomedical Engineering (MIBE), Technical University of Munich, Garching, Germany; [37]German Center for Translational Cancer Research (DKTK), Munich, Germany; [38]The M3 Research Institute, Karls Eberhards Universität Tübingen, Tübingen, Germany; [39]Center for Integrated Oncology Aachen Bonn Cologne Düsseldorf (CIO ABCD), Düsseldorf, Germany; *These Co-last authors contributed equally to this work. §corresponding author.

Correspondence
Tom Luedde, M.D./Ph.D., Department of Gastroenterology, Hepatology and Infectious Diseases, University Hospital Düsseldorf, Medical Faculty at Heinrich-Heine-University, Düsseldorf, Germany.
Email: luedde@hhu.de

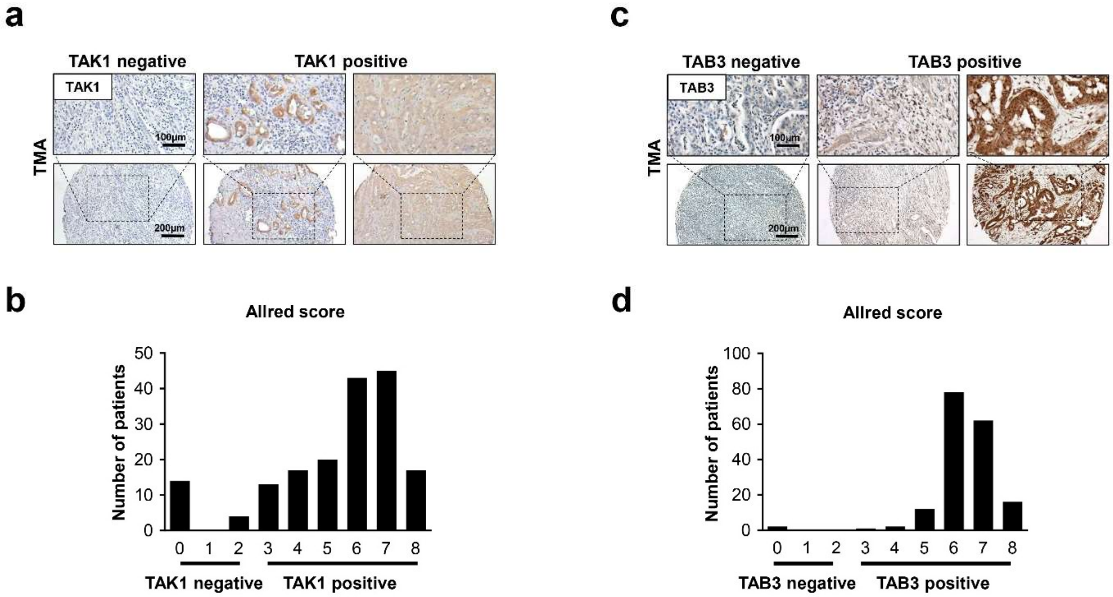

Supplementary Figure 1. Strong expression of TAK1 and TAB3 in human PDAC (related to Figure 1). a-d, Immunohistochemistry (IHC) of TAK1 and TAB3 in samples of a PDAC tissue microarray (TMA) and quantification using the Allred scoring system. (Scores 0-2 = negative, Scores 3-8 = positive). n = 173, biologically independent samples. Source data are provided in the Source Data file.

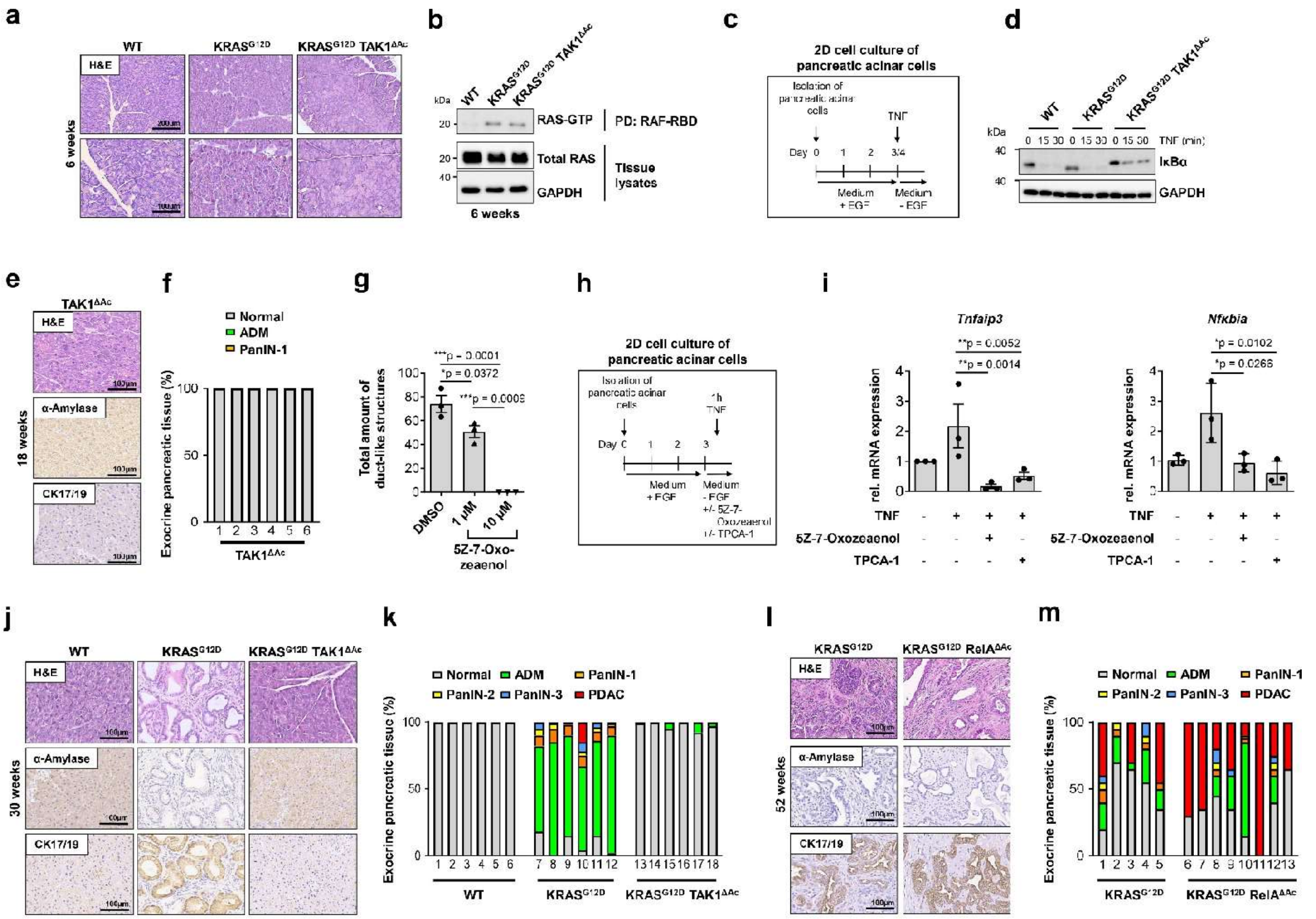
a
WT
KRASG12D
KRASG12D TAK1ΔAc
H&E
6 weeks
200µm
100µm
b
WT
KRASG12D
KRASG12D TAK1ΔAc
kDa
RAS-GTP
PD: RAF-RBD
Total RAS
Tissue lysates
GAPDH
6 weeks
c
2D cell culture of pancreatic acinar cells
Isolation of pancreatic acinar cells
TNF
Day 0 1 2 3/4
Medium + EGF
Medium - EGF
d
kDa
TNF (min)
IκBα
GAPDH
e
TAK1ΔAc
H&E
α-Amylase
CK17/19
18 weeks
f
Normal
ADM
PanIN-1
Exocrine pancreatic tissue (%)
TAK1ΔAc
g
Total amount of duct-like structures
***p = 0.0001
*p = 0.0372
***p = 0.0009
DMSO
1 µM
10 µM
5Z-7-Oxo-zeaenol
h
2D cell culture of pancreatic acinar cells
Isolation of pancreatic acinar cells
1h TNF
Medium + EGF
Medium - EGF +/- 5Z-7-Oxozeaenol +/- TPCA-1
i
Tnfaip3
**p = 0.0052
**p = 0.0014
Nfkbia
*p = 0.0102
*p = 0.0266
rel. mRNA expression
TNF
5Z-7-Oxozeaenol
TPCA-1
j
WT
KRASG12D
KRASG12D TAK1ΔAc
H&E
α-Amylase
CK17/19
30 weeks
k
Normal
ADM
PanIN-1
PanIN-2
PanIN-3
PDAC
Exocrine pancreatic tissue (%)
WT
KRASG12D
KRASG12D TAK1ΔAc
l
KRASG12D
KRASG12D RelAΔAc
H&E
α-Amylase
CK17/19
52 weeks
m
Normal
ADM
PanIN-1
PanIN-2
PanIN-3
PDAC
Exocrine pancreatic tissue (%)
KRASG12D
KRASG12D RelAΔAc

Supplementary Figure 2. KRAS triggers ADM, PanIN and PDAC development that is prevented by *Tak1* deletion (related to Figure 1). a, H&E-Images on pancreatic tissue sections from 6-week-old mice (n = 6). **b,** Immunoblotting analysis of GTP-bound KRAS pull-down (PD) in pancreas tissue lysates from 6-week-old mice. The experiment was done three times with one mouse per genotype. **c,d,** Experimental design to study IκBα-degradation in primary pancreatic acinar cells grown in 2D-cell culture upon TNF-stimulation and subsequent immunoblotting analysis. The experiment was done twice with one mouse per genotype. **e,f**, H&E-, α-Amylase- and CK17/19-images on pancreatic tissue sections from 18-week-old TAK1$^{\Delta Ac}$ mice and quantification of healthy pancreas tissue (Normal), acinar-to-ductal metaplasia (ADM) and pancreatic intraepithelial neoplasia 1 (PanIN-1) (n = 6). **g**, Quantification of the total amount of duct-like structures formed from pancreatic acinar explants isolated from KRAS^{G12D} mice and grown in 3D-collagen matrices ± inhibitors. Experimental design in Figure 1h. Results are expressed as mean ±SEM. The experiment was done with acinar explants from three KRAS^{G12D} mice. *P* value was calculated by ordinary one-way ANOVA (two-tailed) with Tukey's multiple-comparisons test. **h**,**i**, Experimental design to study NF-κB-target gene expression in pancreatic acinar explants isolated from KRAS^{G12D} mice and grown in 2D-cell culture ± inhibitors. After the cells were established from pancreatic acinar explants, they were treated ± inhibitors for 1 h, followed by addition of TNF (100 ng/ml) for another 1h. Relative mRNA expression of *Tnfaip3* and *Nfkbia* was analyzed by qRT-PCR. The experiment was done in triplicate using explants from three KRAS^{G12D} mice. All results were normalized to *β-actin* expression. Results are expressed as mean ±SEM. *P* value was calculated by ordinary one-way ANOVA (two-tailed) with Tukey's multiple-comparisons test. **j**,**k**, H&E-, α-Amylase- and CK17/19-images on pancreatic tissue sections from 30-week-old mice and quantification of the different stages of pancreatic cancer development (n = 6). **l,m,** H&E-, α-Amylase- and CK17/19-images on pancreatic tissue sections from 52-week-old mice and quantification of the different stages of pancreatic cancer development (n = 5 for KRAS^{G12D} and n = 8 for KRAS^{G12D} RelA$^{\Delta Ac}$ mice). Source data are provided in the Source Data file.

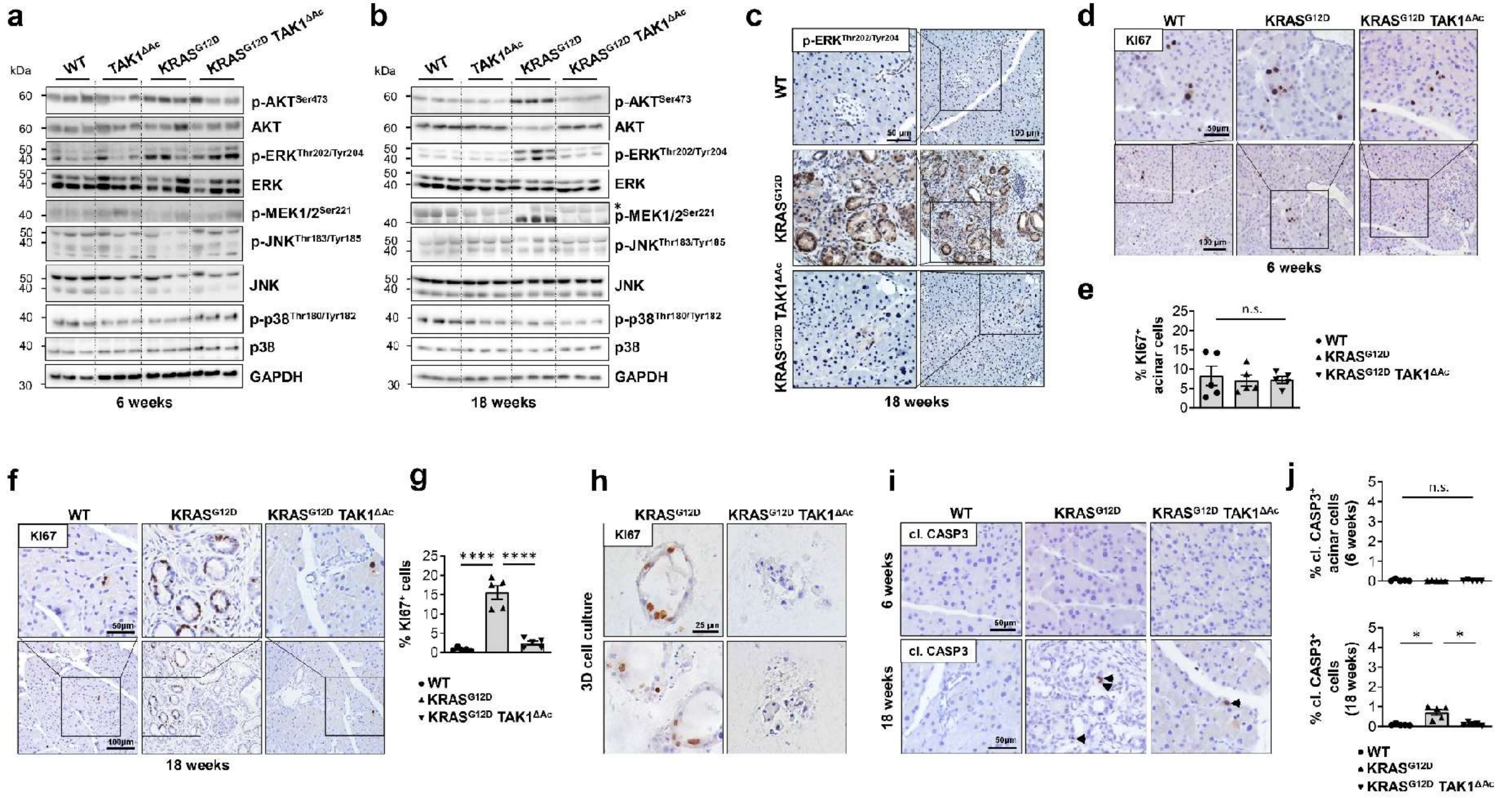
a
b
c
d
e
f
g
h
i
j
kDa
WT
TAK1ΔAc
KRASG12D
KRASG12D TAK1ΔAc
p-AKTSer473
AKT
p-ERKThr202/Tyr204
ERK
p-MEK1/2Ser221
p-JNKThr183/Tyr185
JNK
p-p38Thr180/Tyr182
p38
GAPDH
6 weeks
18 weeks
KI67
% KI67+ acinar cells
n.s.
% KI67+ cells
****
3D cell culture
cl. CASP3
% cl. CASP3+ acinar cells (6 weeks)
% cl. CASP3+ cells (18 weeks)
*

Supplementary Fig. 3. Acinar transdifferentiation causes strong upregulation of KRAS-dependent signaling pathways and triggers proliferation (related to Figure 2). a,b, Immunoblotting analyses of phospho-AKT^{Ser473} (p-AKT ^{Ser473}), AKT, p-ERK$^{Thr202/Tyr204}$, ERK, p-MEK1/2^{Ser221}, p-JNK$^{Thr183/Tyr185}$, JNK, p-p38$^{Thr180/Tyr182}$, p38 and GAPDH (loading control) in lysates of pancreatic tissue of 6- and 18-week-old mice with the indicated genotypes (n = 3 mice per genotype). *, non-specific band. **c**, Representative images of p-ERK IHC on pancreatic tissue sections from 18-week-old mice with the indicated genotype (n = 5 mice per genotype). **d-g**, Representative images of Ki67 IHC and quantification of Ki67$^+$ acinar cells in pancreatic tissue sections from 6-week-old (d,e) or 18-week-old mice (f,g) with the indicated genotypes (n = 5 mice per genotype). Results are expressed as mean ±SEM. *P* value was calculated by ordinary one-way ANOVA (two-tailed) with Tukey's multiple-comparisons test with n.s. = not significant, ****$p < 0.0001$. **h**, Representative images of Ki67 IHC in pancreatic acinar explants from the indicated mice grown in 3D collagen matrices. Experimental design is shown in Figure 1e. The experiment was done with acinar explants from 3 different mice per genotype. **i**,**j**, Representative images of cl. CASP3 IHC and quantification of cl.CASP3$^+$ acinar cells in pancreatic tissue sections from 6-week-old and 18-week-old mice with the indicated genotypes (n = 5 mice per genotype). Results are expressed as mean ±SEM. *P* value was calculated by Kruskal–Wallis test (two-tailed) with Dunn's multiple-comparisons test. n.s. = not significant, 18-week-old-mice: *$p = 0.0154$ WT vs KRAS^{G12D}, *$p = 0.0357$ KRAS^{G12D} vs KRAS^{G12D}/TAK1$^{\Delta Ac}$. Source data are provided in the Source Data file.

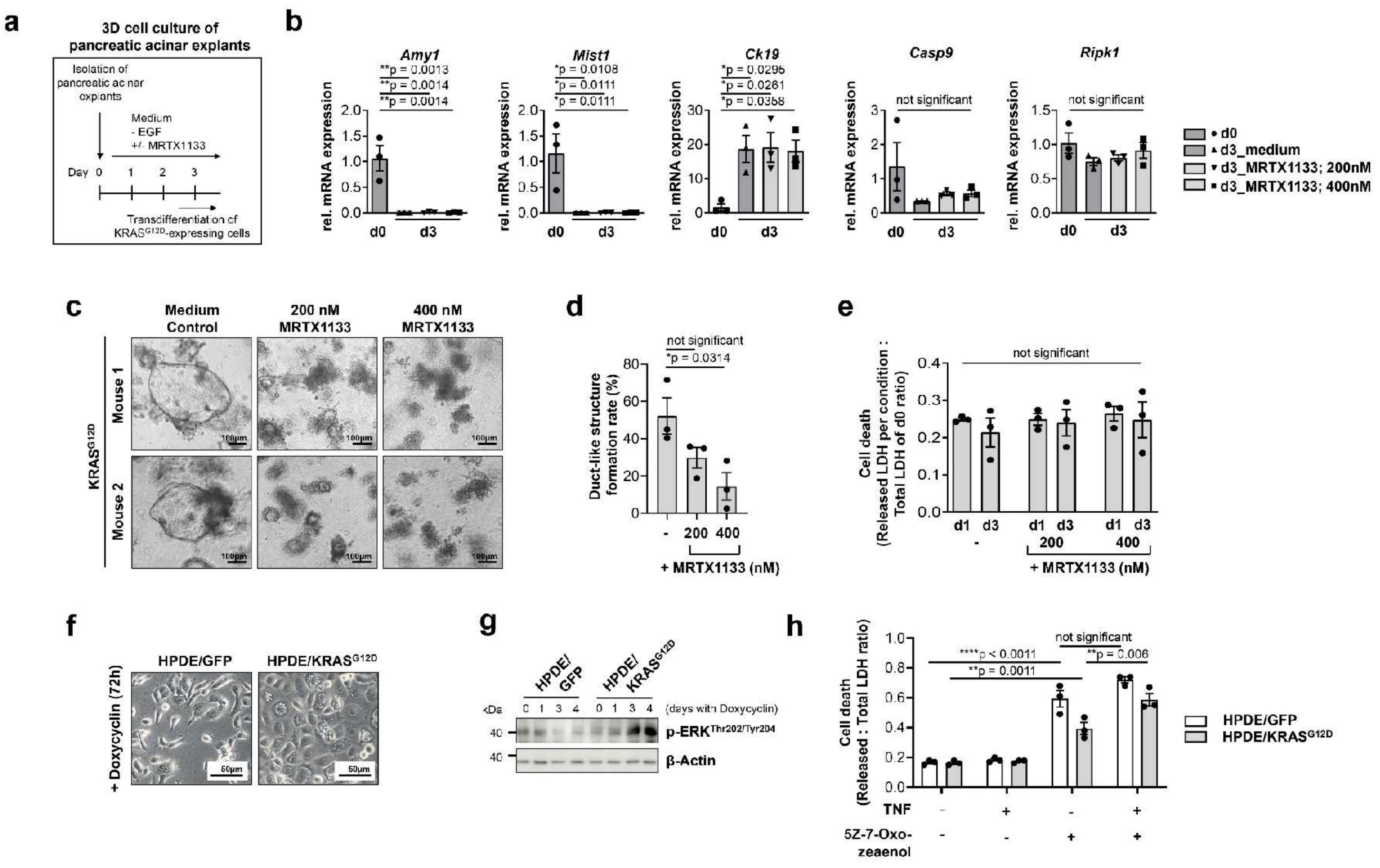
a
3D cell culture of pancreatic acinar explants
Isolation of pancreatic acinar explants
Medium
- EGF
+/- MRTX1133
Day 0 1 2 3
Transdifferentiation of KRAS^{G12D}-expressing cells
b
Amy1
**p = 0.0013
**p = 0.0014
**p = 0.0014
Mist1
*p = 0.0108
*p = 0.0111
*p = 0.0111
Ck19
*p = 0.0295
*p = 0.0261
*p = 0.0358
Casp9
not significant
Ripk1
not significant
rel. mRNA expression
d0
d3
d0
d3_medium
d3_MRTX1133; 200nM
d3_MRTX1133; 400nM
c
Medium Control
200 nM MRTX1133
400 nM MRTX1133
KRAS^{G12D}
Mouse 1
Mouse 2
100µm
d
not significant
*p = 0.0314
Duct-like structure formation rate (%)
- 200 400
+ MRTX1133 (nM)
e
not significant
Cell death (Released LDH per condition : Total LDH of d0 ratio)
d1 d3 d1 d3 d1 d3
- 200 400
+ MRTX1133 (nM)
f
HPDE/GFP
HPDE/KRAS^{G12D}
+ Doxycyclin (72h)
50µm
g
HPDE/ GFP
HPDE/ KRAS^{G12D}
kDa 0 1 3 4 0 1 3 4 (days with Doxycyclin)
40 p-ERK$^{Thr202/Tyr204}$
40 β-Actin
h
****p < 0.0011
**p = 0.0011
not significant
**p = 0.006
Cell death (Released : Total LDH ratio)
TNF - + - +
5Z-7-Oxo-zeaenol - - + +
HPDE/GFP
HPDE/KRAS^{G12D}

Supplementary Figure 4. KRAS activation does not affect the susceptibility of pancreatic ductal cells to cell death (related to Figure 3). a, Experimental design to study duct-like structure formation of $KRAS^{G12D}$ pancreatic acinar cell explants grown in 3D-collagen matrices before (d0) and three days after transdifferentiation into duct-like cells (d3) ± MRTX1133. **b,** qRT-PCR analysis of the mRNA expression at d0 and d3 ± MRTX1133. n = three $KRAS^{G12D}$ mice. All values were normalized to *Sdha* expression. Results are expressed as mean ±SEM. *P* value was calculated by ordinary one-way ANOVA (two-tailed) with Tukey's multiple-comparisons test. **c,d,** Bright-field images and quantification of duct-like structure formation rate at d3 of $KRAS^{G12D}$-expressing pancreatic acinar explants grown in 3D-collagen matrices. The experiment was done with three $KRAS^{G12D}$ mice. Results are expressed as mean ±SEM. *P* value was calculated by ordinary one-way ANOVA (two-tailed) with Tukey's multiple-comparisons test. **e,** Cell death evaluation at d1 and d3 of $KRAS^{G12D}$-expressing pancreatic acinar explant transdifferentiation under the conditions described in a, as assessed by the ratio of released LDH per condition to total LDH at d0. n = three $KRAS^{G12D}$ mice. Results are expressed as mean ±SEM. *P* value was calculated by ordinary one-way ANOVA (two-tailed) with Tukey's multiple-comparisons test. **f,** Bright-field images of HPDE cells treated with 400 ng/ml doxycycline to induce human $KRAS^{G12D}$- or GFP-expression. The experiment was done three times. **g,** Immunoblotting analysis in lysates of HPDE cells treated with 400 ng/ml doxycycline to induce human $KRAS^{G12D}$- or GFP-expression. The experiment was performed once. **h,** Cell death evaluation in HPDE cells treated with 400 ng/ml doxycycline for three days to induce $KRAS^{G12D}$- or GFP-expression, followed by incubation with DMSO, TNF (20 ng/ml), 5Z-7-Oxozeaenol (10 µM) or TNF (20 ng/ml)/5Z-7-Oxozeaenol (10 µM) for additional 48 h. Cell death was assessed by measuring the released LDH-to-total LDH ratio. Results are expressed as mean ±SEM. Each data point represents the result of each individual experiment (n = 3). Each experiment was performed in duplicates. *P* value was calculated by ordinary one-way ANOVA (two-tailed) with Tukey's multiple comparisons test. Source data are provided in the Source Data file.

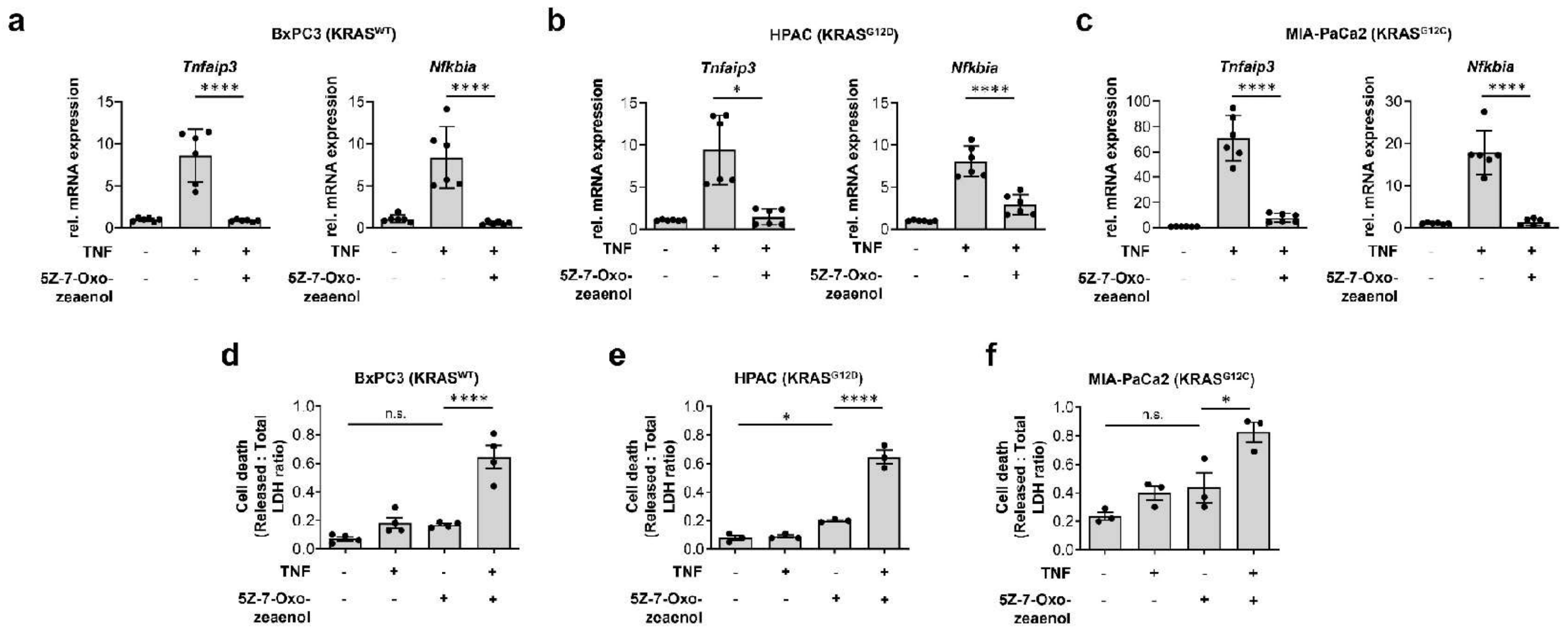
a
BxPC3 (KRAS^WT)
Tnfaip3
Nfkbia
rel. mRNA expression
TNF
5Z-7-Oxo-zeaenol
b
HPAC (KRAS^G12D)
c
MIA-PaCa2 (KRAS^G12C)
d
e
f
Cell death (Released : Total LDH ratio)
n.s.

Supplementary Figure 5. Cell death induction upon TAK1 inhibition in human pancreatic cancer cell lines (related to Figure 4). a-c, Human pancreatic cancer cell lines bearing a different KRAS mutational status (BxPC3: $KRAS^{WT}$, HPAC: $KRAS^{G12D}$ and MIA PaCa-2: $KRAS^{G12C}$) were treated with DMSO (solvent) or 5Z-7-Oxozeaenol (10 µM) for 1 h before TNF (20 ng/ml) was added to the medium for another hour. The relative mRNA expression of the NF-κB target genes *Tnfaip3* and *Nfkbia* was analyzed by qRT-PCR. The experiment was done two times and each column represents the combined results of the two experiments. The individual experiments were run in triplicate. All results were normalized to *β-actin* expression and to the control (DMSO) for each individual experiment, which was set at 1. Results are expressed as mean ±SEM. *P* value was calculated by ordinary one-way ANOVA (two-tailed) with Tukey's multiple comparisons test or Kruskal–Wallis test (two-tailed) with Dunn's multiple-comparisons test. BxPC3 *Tnfaip3:* ****$p < 0.0001$, BxPC3 *Nfkbia:* ****$p < 0.0001$, HPAC *Tnfaip3:* *$p = 0.0105$, HPAC *Nfkbia:* ****$p < 0.0001$, MIA PaCa-2 *Tnfaip3:* ****$p < 0.0001$, MIA PaCa-2 *Nfkbia:* ****$p < 0.0001$. **d-f,** Cell death evaluation in human pancreatic cancer cell lines treated with DMSO (solvent), TNF (20 ng/ml), 5Z-7-Oxozeaenol (10 µM) or TNF (20 ng/ml)/5Z-7-Oxozeaenol (2 µM) for 48 h. Cell death was assessed by measuring the released LDH-to-total LDH ratio. Results are expressed as mean ±SEM. Each data point represents the result of each individual experiment (n = 3). Each treatment per experiment was performed in duplicates. *P* value was calculated by ordinary one-way ANOVA (two-tailed) with Tukey's multiple-comparisons. BxPC3: n.s. = not significant, ****$p < 0.0001$, HPAC: *$p = 0.0411$, ****$p < 0.0001$, MIA PaCa-2: n.s. = not significant, *$p = 0.0159$. Source data are provided in the Source Data file.

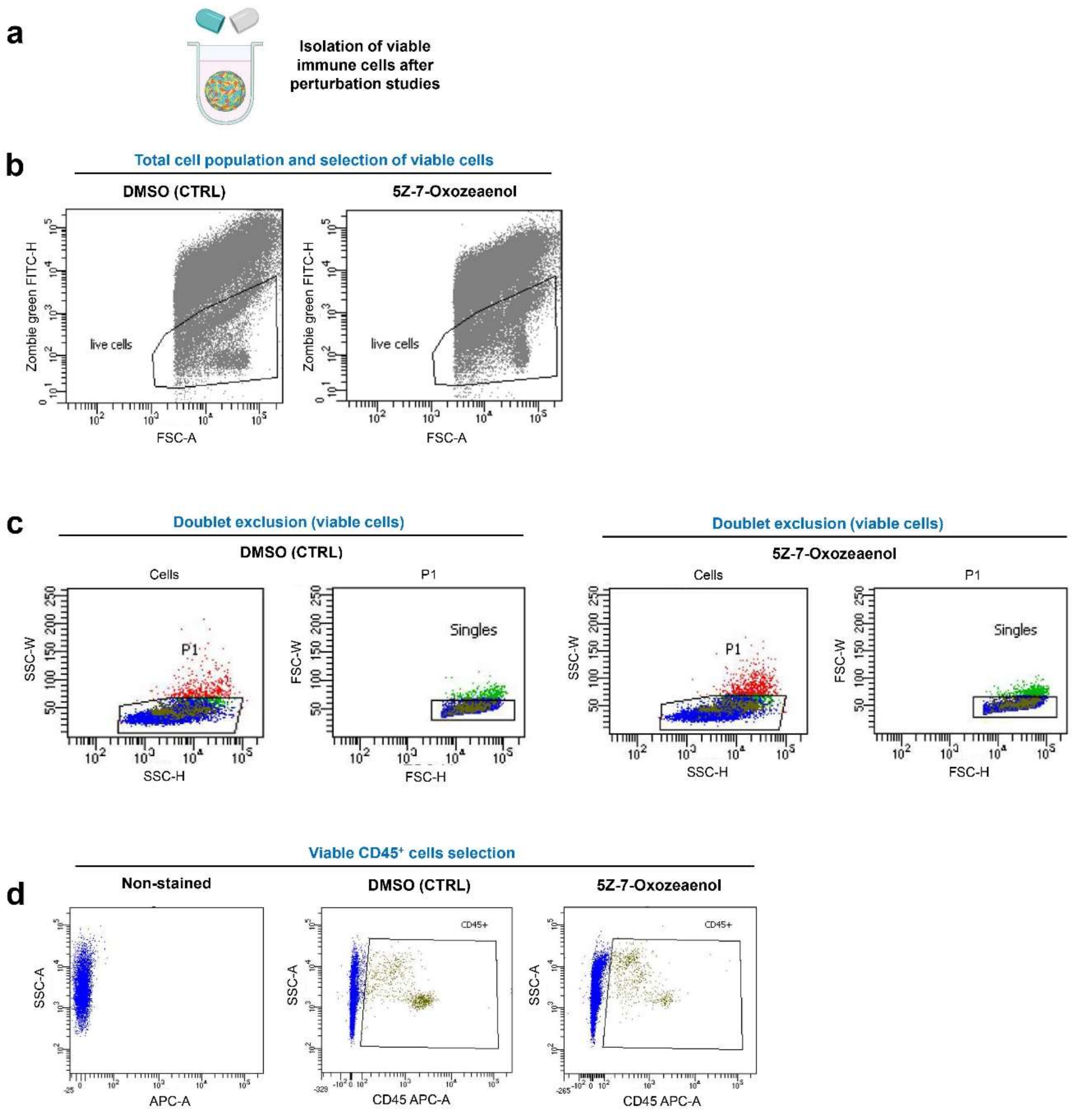

Supplementary Figure 6. Flow cytometry gating strategy for cell sorting (related to Figure 5a-h). a, CD45$^+$ immune cell isolation from tumor patient-derived PDAC tissue by flow cytometry after perturbation studies (Created in BioRender. Schneider, A. (2025) https://BioRender.com/m37a603). After patient-derived tumorspheroid dissociation, total cell population was stained using anti-CD45 antibody coupled with AF647 (APC) and zombie green (ZG, FITC) to detect viable cells. **b,** The gating was performed on total cell population using FITC-H (zombie green)/FSCA dot plots to remove cell debris and select the viable cells. **c,** The viable cells were then gated to isolate "singlets" and exclude "doublets" using plot through SSC-W/SSC-H and FSC-W/FSC-H parameters. **d,** The CD45$^+$ viable immune cells were selected (APC$^+$) using non-stained cells as reference and were sorted in 384 well capture plates for single cell RNA-Seq analysis. Data were analyzed using BD FACSDiva™ Software.

a

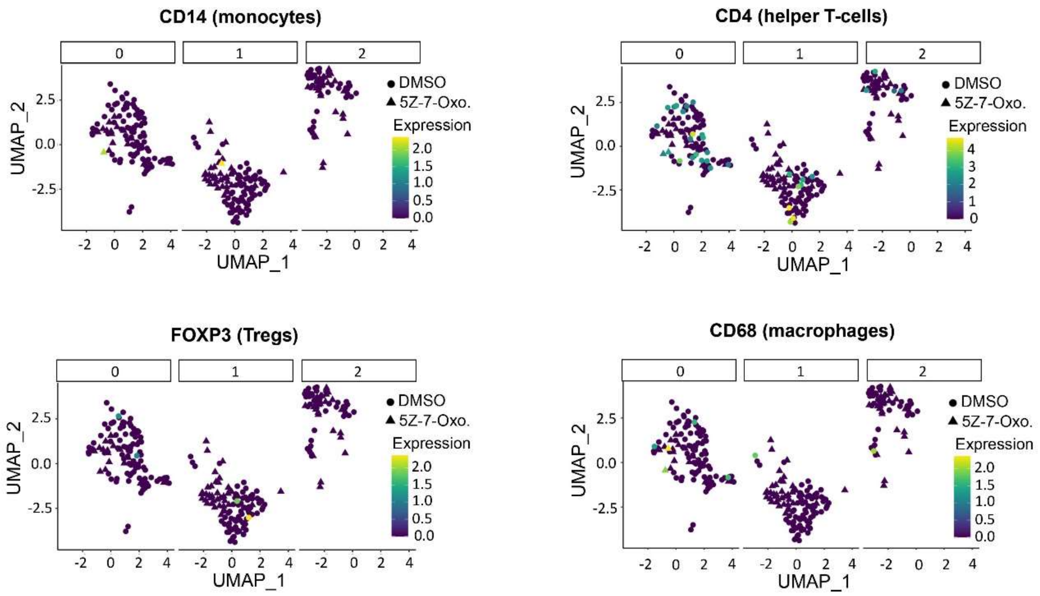

b

GeneSet	Nominal p-value	FDR q-value
GOBP_REGULATION_OF_RESPONSE_TO_STRESS	0.0	0.0
GOBP_LYMPHOCYTE_CHEMOTAXIS	0.0020242915	0.008892668
GOMF_C_C_CHEMOKINE_BINDING	0.0	0.0006526106
GOBP_IMMUNE_RESPONSE	0.0	0.00048324207
GOMF_CYTOKINE_RECEPTOR_ACTIVITY	0.0	0.0
GOMF_IMMUNE_RECEPTOR_ACTIVITY	0.0	0.0
REACTOME_CELLULAR_RESPONSES_TO_STIMULI	0.0	0.0
REACTOME_CYTOKINE_SIGNALING_IN_IMMUNE_SYSTEM	0.0	0.0011187063
REACTOME_INTERFERON_SIGNALING	0.0	0.00005804973
REACTOME_CHEMOKINE_RECEPTORS_BIND_CHEMOKINES	0.0	0.0

Supplementary Figure 7. TAK1-inhibition in PDAC-patient derived tumor-spheroids that were generated by isolation of total cell populations from tumor tissue of one PDAC-patient to assess the immune response (related to Figure 5a-h). a, *UMAP* from DMSO- (circles) or 5Z-7-Oxozeaenol-treated (triangles) PDAC-derived tumor spheroids for the specific markers of the indicated immune cell populations. Each circle/triangle represents one cell. The color bar indicates log2-normalized expression. Experimental setting is shown in Figure 5a. **b,** Summary of Gene Set Enrichment Analysis (GSEA) in cluster 1 of T-cells isolated from 5Z-7-Oxozeaenol (25 µM) vs. DMSO-treated PDAC patient-derived tumor spheroids. Normalized enrichment score (NES) of significantly enriched or suppressed pathways depicted in Fig. 5g, h after 5Z-7-Oxozeaenol (25 µM) treatment (***FDR $q < 0.001$). Source data are provided in the Source Data file.

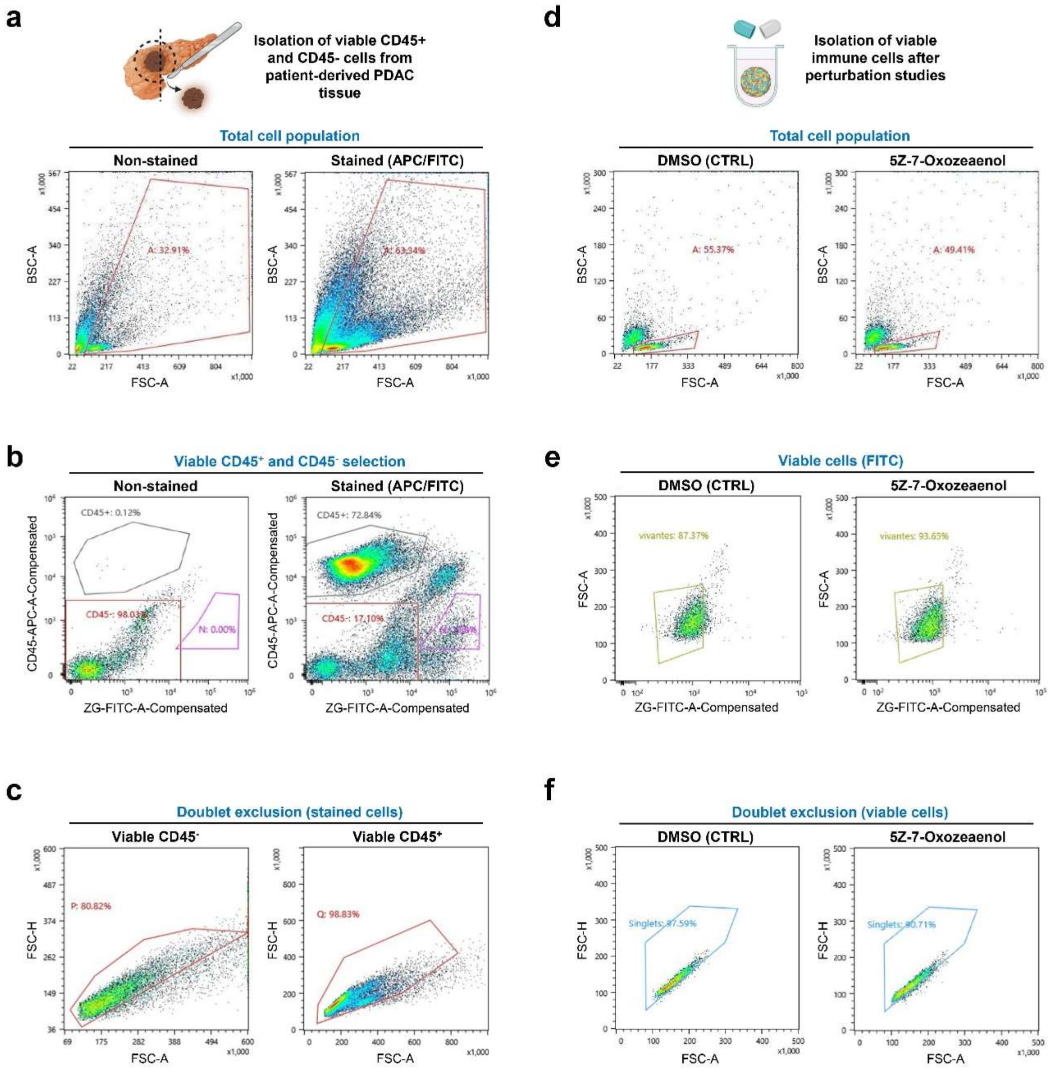

Supplementary Figure 8. Flow cytometry gating strategy for cell sorting (related to Figure 5i-l). a-c, CD45$^+$ immune cell isolation from tumor patient-derived PDAC tissue by flow cytometry (Created in BioRender. Schneider, A. (2025) https://BioRender.com/d09n339). After tissue dissociation, total cell population was stained using anti-CD45 antibody coupled with AF647 (APC) and zombie green (ZG, FITC) to detect viable cells. **a,** The gating was performed on total cell population using FCS/BSC dot plots to remove cell debris. **b,** CD45$^+$ and CD45$^-$ viable cells were selected (APC$^+$ ZG$^-$ and APC$^-$ ZG$^-$) using non-stained cells as reference. **c,** The CD45$^+$ and CD45$^-$ viable cells were then gated to isolate “singlets” and exclude “doublets” using plot through FSC-H and FSC-A parameters. **d-f,** Gating strategy for the sorting of viable immune cells isolated in a-c after perturbation studies (Created in BioRender. Schneider, A. (2025) https://BioRender.com/m37a603). **d,** After perturbation studies, immune cells were harvested and stained with ZG to detect viable cells **e,** Viable cells **f,** The viable cells (ZG$^-$) were then gated to isolate “singlets” and were sorted in 384 well capture plates for single cell RNA-Seq analysis. Data were acquired using Sony SH800 Cell Sorter (Sony).

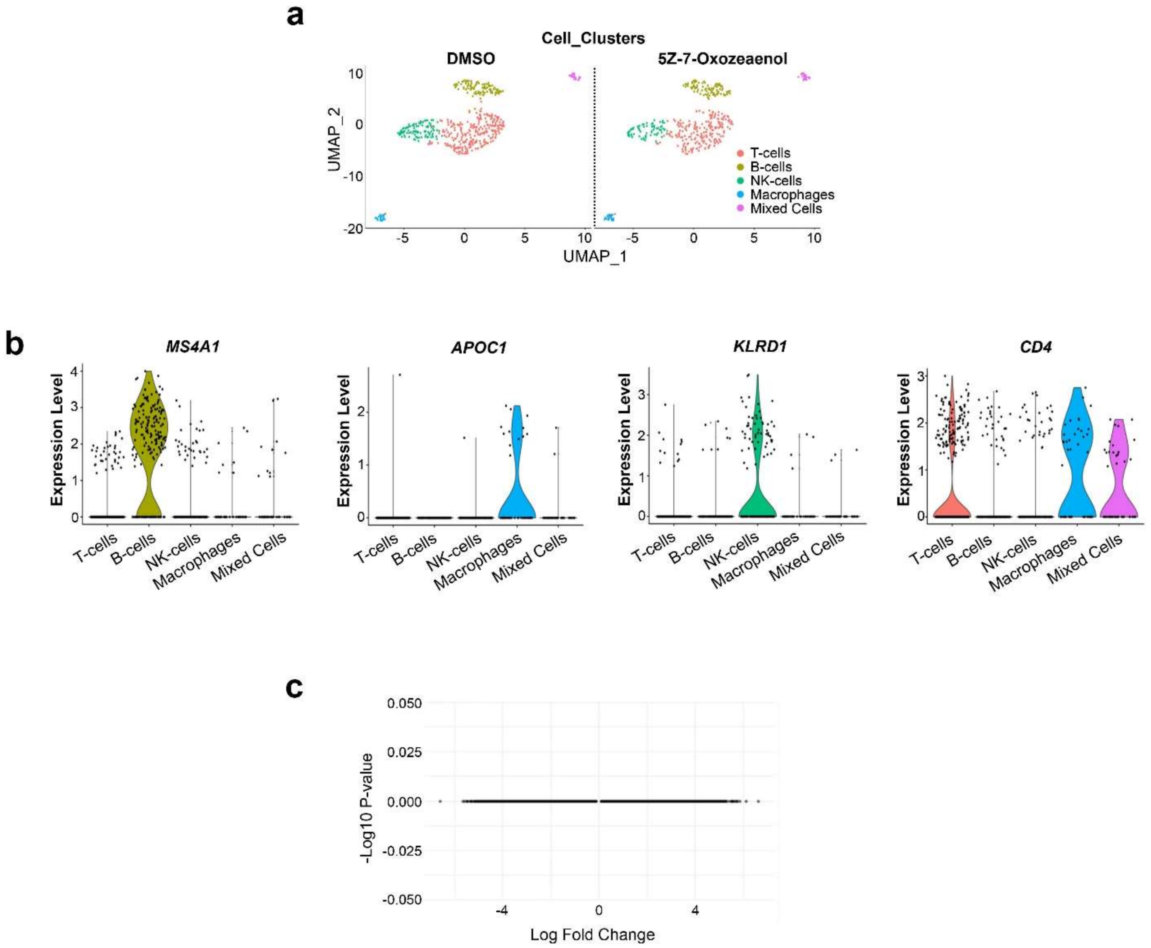

Supplementary Figure 9. TAK1-inhibition on CD45⁺ cell-depleted tumor-spheroids that were generated by isolation and *in vitro* reconstitution from tumor tissue of one PDAC-patient to assess the immune response (related to Figure 5i-l). a, *UMAP* of single-cell transcriptome clustering of PDAC-derived immune cells incubated with the supernatant of DMSO- or 5Z-7-Oxozeaenol-treated PDAC-derived tumor spheroids showing five distinct cell populations (T-cells, B-cells, NK-cells, macrophages and mixed-cells). Each dot represents one cell. Experimental setting is shown in Figure 5i. **b**, Violin plot of single-cell transcriptome expression levels of immune cell-specific markers. The immune cells were incubated with the supernatant of DMSO- or 5Z-7-Oxozeaenol-treated PDAC-derived tumor spheroids from the same PDAC-patient. Each dot represents one cell. Experimental setting is shown in Figure 5i. **c**, Volcano plot of differentially expressed genes of PDAC-derived immune cells incubated with the supernatant of DMSO- or 5Z-7-Oxozeaenol-treated PDAC-derived tumor spheroids showing no differences in gene expression. x axis represents magnitude of the difference as determined by log fold change, y axis measures significance using -log10 p-value. Source data are provided in the Source Data file.

Supplementary Table 1. Basic characteristics of the PDAC-patient samples

Variable	Proportion among patients with allred **score 0** n = 14	Proportion among patients with allred **score 2** n = 4	Proportion among patients with allred **score 3** n = 13	Proportion among patients with allred **score 4** n = 17	Proportion among patients with allred **score 5** n = 20	Proportion among patients with allred **score 6** n = 43	Proportion among patients with allred **score 7** n = 45	Proportion among patients with allred **score 8** n = 17
**Age (Mean, SD)**	58.7 (9.1)	63.8 (9.5)	64.3 (9.6)	64.3 (9.5)	62.3 (11.1)	65.0 (9.0)	63.6 (8.2)	64.9 (12.8)
**Women**	42.9	50.0	30.8	29.4	70.0	55.8	46.7	41.2
**Men**	57.1	50.0	69.2	70.6	30.0	44.2	53.3	58.8
**Tumor grade 1**	7.1	0.0	0.0	11.8	0.0	2.3	2.2	0.0
**Tumor grade 2**	85.7	50.0	69.2	58.8	80.0	67.4	51.1	35.3
**Tumor grade 3**	7.1	50.0	30.8	29.4	20.0	30.2	46.7	64.7

Porportion of patients in %, unless otherwise indicated. SD: Standard deviation

Supplementary Table 2. Basic characteristics of the patient-derived organoid (PDO) lines

Patient-derived organoid (PDO) lines	KRAS status (Sanger-Seq)	Source	Gender	Age at the time of diagnosis	p Staging	Grading
Patient 1	G12D	Surgery	male	60-70	ypT3ypN1(3/23)Pn1	-
Patient 2	G12A	FNB	female	60-70	-	-
Patient 3	G12D	Surgery	male	70-80	pT3pN1(1/35)Pn1	G2-3

FNB: fine-needle biopsy

Supplementary Table 3. Basic characteristics of the patient-derived spheroids that were generated by isolation of total cell populations from tumor tissue of one PDAC-patient to assess the immune response

Patient-derived spheroids	Source	Gender	Age at the time of diagnosis	p Staging
Patient	Surgery	female	60-70	ypT2N0

Supplementary Table 4. T lymphocyte populations per cluster detected in PDAC patient-derived tumor-spheroids

T lymphocyte populations	Cluster_0		
	total amount (n)	DMSO-treated (%)	5Z-7-Oxozeaenol-treated (%)
CD3E	75	86,7 (n = 65)	13,3 (n = 10)
CD4	21	90,5 (n = 19)	9,5 (n = 2)
CD8A	39	100,0 (n = 39)	0,0 (n = 0)
CD44	75	88,0 (n = 66)	12,0 (n = 9)

T lymphocyte populations	Cluster_1		
	total amount (n)	DMSO-treated (%)	5Z-7-Oxozeaenol-treated (%)
CD3E	42	81,0 (n = 34)	19,0 (n = 8)
CD4	8	100,0 (n =8)	0,0 (n = 0)
CD8A	16	87,5 (n = 14)	12,5 (n = 2)
CD44	47	78,7 (n = 37)	21,3 (n = 10)

T lymphocyte populations	Cluster_2		
	total amount (n)	DMSO-treated (%)	5Z-7-Oxozeaenol-treated (%)
CD3E	22	81,8 (n = 18)	18,2 (n = 4)
CD4	4	100,0 (n = 4)	0,0 (n = 0)
CD8A	19	73,7 (n = 14)	26,3 (n = 5)
CD44	23	82,6 (n = 19)	17,4 (n = 4)

Supplementary Table 5. Basic characteristics of the patient-derived spheroids that were generated by isolation and *in vitro* reconstitution from tumor tissue of one PDAC-patient to assess the immune response

Patient-derived spheroids	Source	Gender	Age at the time of diagnosis	p Staging
Patient	Surgery	female	60-70	pT1cN0

Supplementary Table 6. qRT-PCR primer sequences

**Mouse:**	
Beta-actin forward	CGG TTC CGA TGC CCT GAG GCT CTT
Beta-actin reverse	CGT CAC ACT TCA TGA TGG AAT TGA
Nfkbia forward	TGA AGG ACG AGG AGT ACG AGC
Nfkbia reverse	TTC GTG GAT GAT TGC CAA GTG
Tnfaip3 forward	GAA CAG CGA TCA GGC CAG G
Tnfaip3 reverse	GGA CAG TTG GGT GTC TCA CAT T
Ripk1 forward	GAC TGT GTA CCC TTA CCT CCG A
Ripk1 reverse	CAC TGC GAT CAT TCT CGT CCT G
Ripk3 forward	GAA GAC ACG GCA CTC CTT GGT A
Ripk3 reverse	CTT GAG GCA GTA GTT CTT GGT GG
Mlkl forward	CTG AGG GAA CTG CTG GAT AGA G
Mlkl reverse	CGA GGA AAC TGG AGC TGC TGA T
Casp8 forward	ATG GCT ACG GTG AAG AAC TGC G
Casp8 reverse	TAG TTC ACG CCA GTC AGG ATG C
Casp3 forward	GGA GTC TGA CTG GAA AGC CGA A
Casp3 reverse	CTT CTG GCA AGC CAT CTC CTC A
Amy1 forward	CAG CAC TTG TGG CAA TGA CTG G
Amy1 reverse	GCA AAA GGC TGA CCA TTG ACG AC
Mist-1 forward	AGG AGA GCA GAC ACC TGA CAG A
Mist-1 reverse	CCG CCT CTG AAC ACT GTT CTC C
Ck19 forward	GGG GGT TCA GTA CGC ATT GG
Ck19 reverse	GAG GAC GAG GTC ACG AAG C
Casp9 forward	GGC TGT TAA ACC CCT AGA CCA
Casp9 reverse	TGA CGG GTC CAG CTT CAC TA
Sdha forward	TGG GGA GTG CCG TGG TGT CA
Sdha reverse	GTG CCG TCC CCT GTG CTG GT
**Human:**	
Beta-actin forward	CAT GTA CGT TGC TAT CCA GGC
Beta-actin reverse	CTC CTT AAT GTC ACG CAC GAT
IκBα forward	CTA CCA ACT ACA ATG GCC AC
IκBα reverse	GGC TCC TGA GCA TTG ACA TC
A20 forward	CAG AAG AGC AAC TGA GAT CG
A20 reverse	GAC ACT CCA TGC AGA GCT CC

a

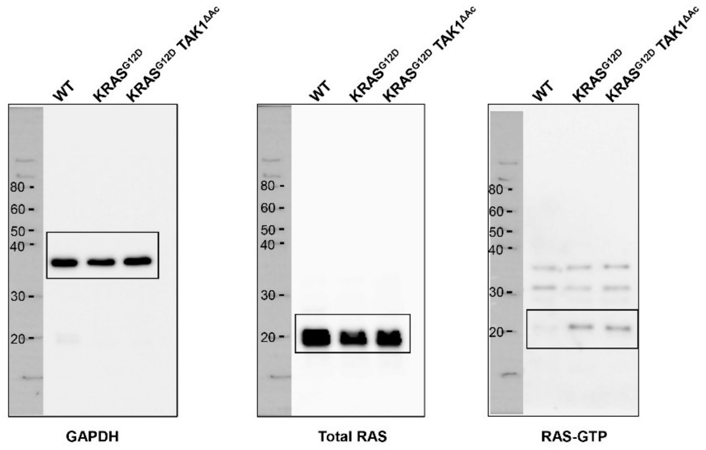

b

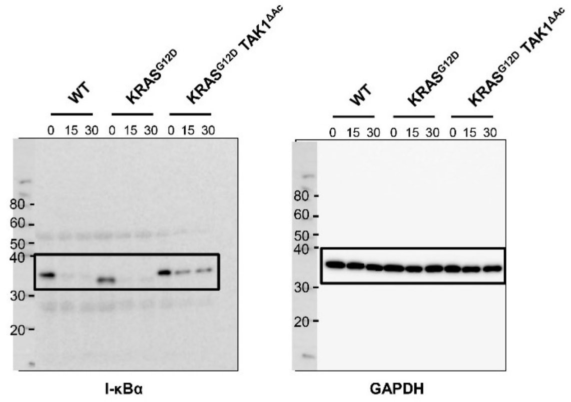

Related to Supplementary Figure 2b. Uncropped western blot scans. a, Immunoblotting analysis of GTP-bound KRAS pull-down (PD) in pancreas tissue lysates from 6-week-old mice. The experiment was done three times with one mouse per genotype (related to Suppl. Figure 2b). **b,** Immunoblotting analysis to study IκBα-degradation in primary pancreatic acinar cells grown in 2D-cell culture upon TNF-stimulation. The experiment was done twice with one mouse per genotype (related to Suppl. Figure 2d). Source data are provided in the Source Data file.

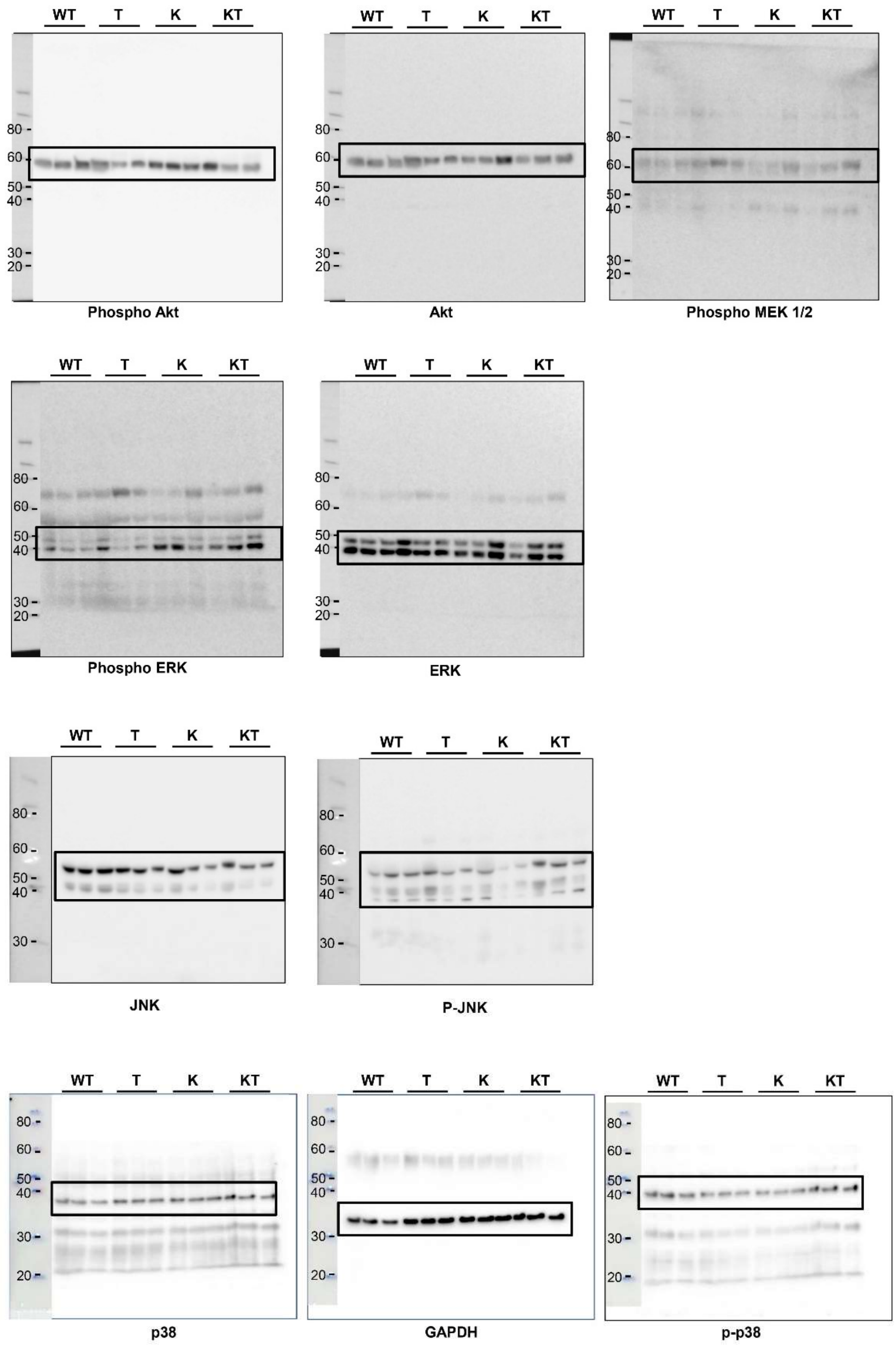

Related to Supplementary Fig. 3a. Uncropped western blot scans. Immunoblotting analyses of phospho-AKT^{Ser473} (p-AKT ^{Ser473}), AKT, p-ERK$^{Thr202/Tyr204}$, ERK, p-MEK1/2^{Ser221}, p-JNK$^{Thr183/Tyr185}$, JNK, p-p38$^{Thr180/Tyr182}$, p38 and GAPDH (loading control) in lysates of pancreatic tissue of 6-week-old mice with the indicated genotypes (n = 3 mice per genotype). Source data are provided in the Source Data file.

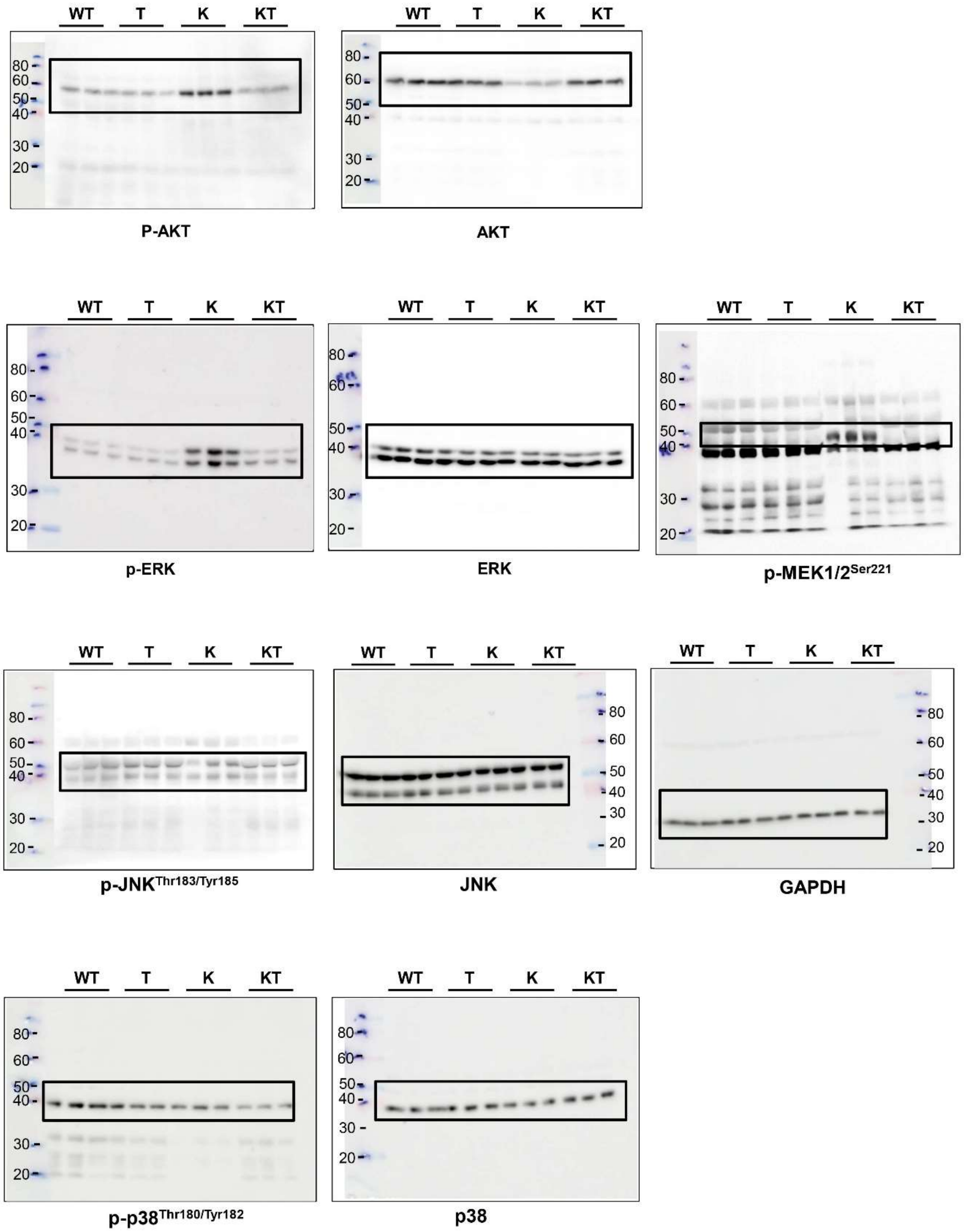

Related to Supplementary Fig. 3b. Uncropped western blot scans. Immunoblotting analyses of phospho-AKT^{Ser473} (p-AKT ^{Ser473}), AKT, p-ERK$^{Thr202/Tyr204}$, ERK, p-MEK1/2^{Ser221}, p-JNK$^{Thr183/Tyr185}$, JNK, p-p38$^{Thr180/Tyr182}$, p38 and GAPDH (loading control) in lysates of pancreatic tissue of 18-week-old mice with the indicated genotypes (n = 3 mice per genotype). Source data are provided in the Source Data file.

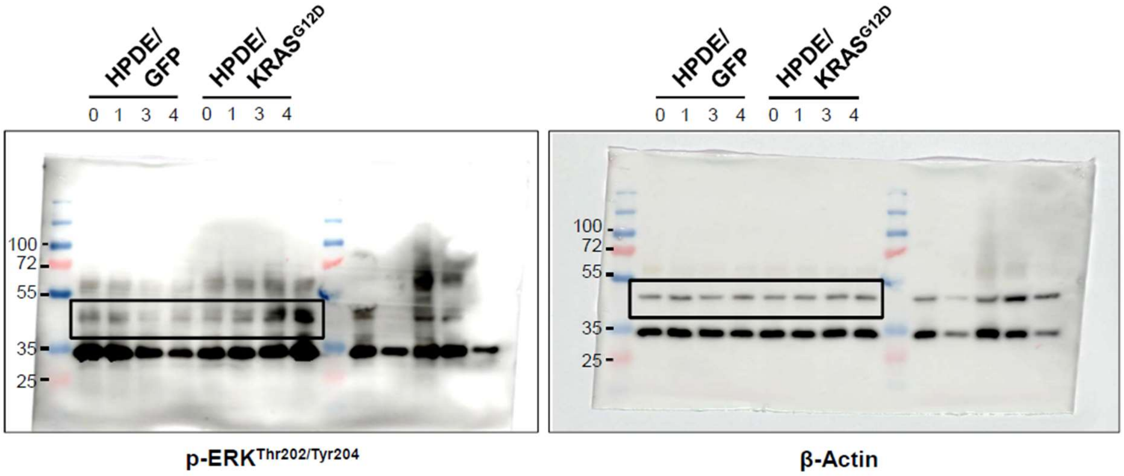

Related to Supplementary Fig. 4g. Uncropped western blot scans. Immunoblotting analysis of p-ERK$^{Thr202/Tyr204}$ and β-Actin (loading control) in lysates of HPDE cells treated with 400 ng/ml doxycycline to induce the expression of human KRAS^{G12D} or GFP for the indicated time. Source data are provided in the Source Data file.
