# Supplementary material for: A decision point between transdifferentiation and programmed cell death priming controls KRAS-dependent pancreatic cancer development
Source: Nat Commun. 2025 Feb 19;16:1765. doi: 10.1038/s41467-025-56493-7 (PMC11839950; doi:10.1038/s41467-025-56493-7)
Supplement: Supplementary file 2 — Description of Additional Supplementary Files [file 41467_2025_56493_MOESM2_ESM.pdf]

Description of Additional Supplementary Files

File Name: Supplementary Data 1
Description: Summary of Gene Set Enrichment Analysis (GSEA) in cluster 1 of T-cells isolated from 5Z-7-Oxozeaenol (25 μM) vs. DMSO-treated PDAC patient-derived tumor spheroids. Normalized enrichment score (NES) of significantly enriched or suppressed pathways depicted in Fig. 5g, h after 5Z-7-Oxozeaenol (25 μM) treatment (***FDR q < 0.001).

File Name: Supplementary Data 2
Description: Gene Set Enrichment Analysis (GSEA) of PDAC-derived T cells incubated with the conditioned medium from DMSO- or 5Z-7-Oxozeaenol-treated tumor spheroids. NES was non-significantly altered (FDR > 0.05) in all pathways that were affected after 5Z-7-Oxozeaenol treatment in Fig. 5g-h.
