# Supplementary material for: A decision point between transdifferentiation and programmed cell death priming controls KRAS-dependent pancreatic cancer development
Source: Nat Commun. 2025 Feb 19;16:1765. doi: 10.1038/s41467-025-56493-7 (PMC11839950; doi:10.1038/s41467-025-56493-7)
Supplement: Supplementary file 5 — Reporting Summary [file 41467_2025_56493_MOESM5_ESM.pdf]

nature portfolio

Corresponding author(s): Tom Luedde

Last updated by author(s): 2025.01.20

Reporting Summary

Nature Portfolio wishes to improve the reproducibility of the work that we publish. This form provides structure for consistency and transparency in reporting. For further information on Nature Portfolio policies, see our Editorial Policies and the Editorial Policy Checklist.

Statistics

For all statistical analyses, confirm that the following items are present in the figure legend, table legend, main text, or Methods section.

n/a	Confirmed	
☐	☒	The exact sample size (*n*) for each experimental group/condition, given as a discrete number and unit of measurement
☐	☒	A statement on whether measurements were taken from distinct samples or whether the same sample was measured repeatedly
☐	☒	The statistical test(s) used AND whether they are one- or two-sided<br>*Only common tests should be described solely by name; describe more complex techniques in the Methods section.*
☒	☐	A description of all covariates tested
☐	☒	A description of any assumptions or corrections, such as tests of normality and adjustment for multiple comparisons
☐	☒	A full description of the statistical parameters including central tendency (e.g. means) or other basic estimates (e.g. regression coefficient) AND variation (e.g. standard deviation) or associated estimates of uncertainty (e.g. confidence intervals)
☐	☒	For null hypothesis testing, the test statistic (e.g. *F*, *t*, *r*) with confidence intervals, effect sizes, degrees of freedom and *P* value noted<br>*Give P values as exact values whenever suitable.*
☒	☐	For Bayesian analysis, information on the choice of priors and Markov chain Monte Carlo settings
☒	☐	For hierarchical and complex designs, identification of the appropriate level for tests and full reporting of outcomes
☒	☐	Estimates of effect sizes (e.g. Cohen's *d*, Pearson's *r*), indicating how they were calculated

Our web collection on statistics for biologists contains articles on many of the points above.

Software and code

Policy information about availability of computer code

Data collection	Real-Time PCR: QuantStudioTM Real-Time PCR software v1.1<br>ELISA-Reader: i-control 2.0 (for infinite reader)<br>aCGH: Feature Extraction Software (Agilent)<br>Western blot band intensity: IQ800-Control software and Image Quant LAS 4000 software<br>FACS: Sony SH800 cell sorter software V2.1.5
Data analysis	representative H/E and IHC images: Aperio ImageScope software<br>statistics: Graphpad Prism 9<br>Real-Time PCR: QuantStudioTM Real-Time PCR software v1.1<br>scRNA-Seq clustering and differential gene expression analysis: Seurat (3.2.2) R toolkit, R (3.5.3) with Seurat and package "RaceID"<br>scRNA-Seq reads aligned to the human hG19 UCSC reference: HiSat2<br>aCGH: R statistical platform (R Development Core Team. R: A language and environment for statistical computing. R Foundation for Statistical Computing, Vienna, Austria. ISBN 3-900051-07-0, URL: http://www.R-project.org/)<br>FACS: Sony SH800 cell sorter software V2.1.5

For manuscripts utilizing custom algorithms or software that are central to the research but not yet described in published literature, software must be made available to editors and reviewers. We strongly encourage code deposition in a community repository (e.g. GitHub). See the Nature Portfolio guidelines for submitting code & software for further information.

Data

Policy information about availability of data

All manuscripts must include a data availability statement. This statement should provide the following information, where applicable:

- Accession codes, unique identifiers, or web links for publicly available datasets
- A description of any restrictions on data availability
- For clinical datasets or third party data, please ensure that the statement adheres to our policy

The aCGH data generated in this study have been deposited at NCBI Gene Expression Omnibus database https://www.ncbi.nlm.nih.gov/geo/query/acc.cgi?acc=GSE282891 under accession number GSE282891. 'The RNA-seq data generated in this study are available at NCBI Gene Expression Omnibus database https://www.ncbi.nlm.nih.gov/geo/query/acc.cgi?acc=%20GSE223135 under the accession number GSE223135 or https://www.ncbi.nlm.nih.gov/geo/query/acc.cgi?acc=%20GSE275488 under accession number GSE275488. Source data are provided with this paper. The remaining data are available within the Article, Supplementary Information or Source Data file.

Research involving human participants, their data, or biological material

Policy information about studies with human participants or human data. See also policy information about sex, gender (identity/presentation), and sexual orientation and race, ethnicity and racism.

Reporting on sex and gender	no sex-and gender-based analyses have been performed. This study did not involve sex or gender research.
Reporting on race, ethnicity, or other socially relevant groupings	no race, ethnicity, or other socially relevant groupings have been performed. This study did not involve race, ethnicity, or other socially relevant groupings research.
Population characteristics	Human PDAC specimens were aquired from patients who were diagnosed with PDAC and collected at the National Center for Tumor Diseases Heidelberg (for TMA analysis), the Technical University Munich (for PDO generation) or at the Strasbourg University Hospitals (for spheroid generation). All patients received standard surgical resection or fine-needle biopsy. Paraffin embedded tissues were preprocessed by a pathologist after surgical resection or fine-needle biopsy and confirmed as PDAC prior to further investigation. Patients have given their informed consent without being paid. The gender of the participants was determined on the basis of self-reports. No gender was excluded. Basic characteristics of the patients can be found in Supplementary Table 1, 2, 3 and 5.
Recruitment	Human PDAC specimens were aquired from patients who were diagnosed with PDAC and collected at the National Center for Tumor Diseases Heidelberg (for TMA analysis), the Technical University Munich (for PDO generation) or at the Strasbourg University Hospitals (for spheroid generation). All patients received standard surgical resection or fine-needle biopsy. Paraffin embedded tissues were preocessed by a pathologist after surgical resection or fine-needle biopsy and confirmed as PDAC prior to further investigation. Patients have given their informed consent without being paid. The gender of the participants was determined on the basis of self-reports. No gender was excluded.
Ethics oversight	TMA: Human pancreatic tissue samples were provided by the tissue bank of the National Center for Tumor Diseases Heidelberg (NCT, Heidelberg, Germany) in agreement with the regulations of the tissue bank and local Ethics Committee of the University of Heidelberg approval (no. 206/2005). The project conducted in accordance with the ethical standards laid down in the Declaration of Helsinki.<br><br>Spheroids: Human pancreatic tissues and serum were obtained from diseased patients undergoing surgery with informed consent from all patients for de-identified use at the Strasbourg University Hospitals, University of Strasbourg, France (DC-2016-2616 and RIPH2 LivMod IDRCB 2019-A00738-49, ClinicalTrial NCT04690972). The protocols were approved by the local Ethics Committee of the University of Strasbourg Hospitals ethical committee. All material was collected during a medical procedure strictly performed within the frame of the medical treatment of the patient. Informed consent is provided according to the Declaration of Helsinki. Detailed patient information and informed consent procedures are implemented by the Strasbourg University Hospital Biological Resources Center (HUS CRB). Patients were given an information sheet which outlines that their left-over biological material that was collected during their medical treatment is requested for research purposes. All patients received and signed an informed consent form (protocols DC-2016-2616 and RIPH2 LivMod IDRCB 2019-A00738-49 ClinicalTrial NCT04690972). The identity of the patients was protected by internal coding.<br><br>PDO: All patients enrolled in the study gave consent prior to PDO generation based on the institutional review board (IRB) project-number 207/15 of the Technical University Munich. Experimental procedures involving human subjects were performed in agreement with the ethical principles for medical research as defined by the WMA Declaration of Helsinki and the Department of Health and Human Services Belmont Report.

Note that full information on the approval of the study protocol must also be provided in the manuscript.

Field-specific reporting

Please select the one below that is the best fit for your research. If you are not sure, read the appropriate sections before making your selection.

☒ Life sciences ☐ Behavioural & social sciences ☐ Ecological, evolutionary & environmental sciences

For a reference copy of the document with all sections, see nature.com/documents/nr-reporting-summary-flat.pdf

Life sciences study design

All studies must disclose on these points even when the disclosure is negative.

Sample size	No statistical methods were used to predetermine sample sizes for in vitro experiments. Sample sizes were chosen in order to be able to perform statistical analyses, as is standard in the field.<br><br>For in vivo experiments, littermates carrying the respective loxP-flanked alleles but lacking expression of Cre recombinase were used as wild-type (WT) controls. Age-, gender-, and equal average tumour volume-matched mice were randomly allocated to different experimental groups based on their genotypes and experiments were not blinded. Both male and female mice are included in all groups. A precalculation of the in vivo mouse sample sizes was performed and approved to ensure an optimal balance between the animal welfare guidelines as well as a reasonable sample number for the experiments. Sample sizes and mice age is indicated in the figure legends.
Data exclusions	No data were excluded for all the analyses described.
Replication	All experiments were repeated at least twice or performed with independent samples. The two scRNA-seq analyses were each carried out with human tumor spheroids derived from one PDAC patient. The exact repeat times of experiments and sample numbers are indicated in the figure legends.
Randomization	Age-, gender-, and equal average tumour volume-matched mice were randomly allocated to different experimental groups based on their genotypes. For experiments other than mice, we did not carry out randomization because it is either irrelevant or not applicable to these studies.
Blinding	Investigators were not blinded to the group allocation except for microscopic analysis of IHC staining results. For other experiments, the investigators were not blinded since analysis relied on unbiased measurements of quantitative parameters. Standardized procedures for the data collection and analysis were used to prevent bias.

Reporting for specific materials, systems and methods

We require information from authors about some types of materials, experimental systems and methods used in many studies. Here, indicate whether each material, system or method listed is relevant to your study. If you are not sure if a list item applies to your research, read the appropriate section before selecting a response.

Materials & experimental systems

n/a	Involved in the study	
☐	☒	Antibodies
☐	☒	Eukaryotic cell lines
☒	☐	Palaeontology and archaeology
☐	☒	Animals and other organisms
☒	☐	Clinical data
☒	☐	Dual use research of concern
☒	☐	Plants

Methods

n/a	Involved in the study	
☒	☐	ChIP-seq
☐	☒	Flow cytometry
☒	☐	MRI-based neuroimaging

Antibodies

Antibodies used	Western blot:<br>primary antibodies (1:1000):<br>anti-beta-actin (Sigma, cat no. A2066, lot: 0000182447)<br>anti-cl. CASP3 Asp175 (Cell Signaling, cat no. 9661, lot: 47)<br>anti-p-ERK Thr202/Tyr204 (Cell Signaling, cat no. 4377, clone: 197G2, lot: 12)<br>anti-ERK (Cell Signaling, cat no. 4695, clone: 137F5, lot: 5)<br>anti-p-Akt Ser473 (Cell Signaling, cat no. 4060, clone: D9E, lot: 27)<br>anti-Akt (Cell Signaling, cat no. 4685, clone: 11E7, lot: 6)<br>anti-JNK1/2 (Cell Signaling, cat no. 9258, clone: 56G8, lot: 11)<br>anti-p-JNK1/2 Thr183/Tyr185 (Cell Signaling, cat no. 4668, clone: 81E11, lot: 2)<br>anti-p38 (Cell Signaling, cat no. 9212, lot: 12)<br>anti-p-p38 Thr180/Tyr182 (Cell Signaling, cat no. 9215, clone: 3D7, lot: 7)<br>anti-p-MEK1/2 Ser221 (Cell Signaling, cat no. 2338, clone: 166F8, lot: 9)<br>anti-I-kappaBalpha (Cell Signaling, cat no. 4812, clone: 44D4, lot: 13)<br>anti-p-MLKL Ser345 (Cell Signaling, cat no. 37333, clone: D6E3G, lot: 2)<br>anti-RIPK3 (Novus, cat no. Img-5523-1, lot: 8337-1803)<br>anti-MLKL (Merck Millipore, cat no. MABC604, clone: 3H1)<br>anti-HSP90 (Enzo, cat no. ADI-SPA-830-D, clone: AC88, lot: 02011766)<br>anti-GAPDH (ABD Serotec, cat no. MCA4739, clone: 6C5, lot: 161665)

secondary antibodies (1:5000):
anti-rabbit-HRP (Cytiva, cat no. NA934)
anti-mouse-HRP (Cytiva, cat no. NA931)

RAS activation assay:
anti-RAS (Merck Millipore, cat no. 05-516, clone: RAS10, 1:1000)

Flow cytometry:
REA Control (S) anti-human APC (Miltenyi Biotec, cat no. 130-113-434, clone: REA293, lot: 5220405741, 1:50)
CD45 Antibody, anti-human APC (Miltenyi Biotec, cat no. 130-110-633, clone: REA747, lot: 5220310224, 1:50)

Immunohistochemistry:
anti-α-Amylase (Cell Signaling, cat no. 3796, clone: D55H10, lot: 3, 1:2000)
anti-Keratin 17/19 (CK17/19) (Cell Signaling, cat no. 3984, clone: D32D9, lot: 1, 1:1000)
anti-SOX9 (Merck Millipore, cat no. AB5535, lot: 2724407, 1:2000)
anti-KI67 (Thermo Scientific, cat no. RM-9106-S, clone: SP6, lot: 9106R0723, 1:2000 mouse)
anti-Ki-67 (Abcam, cat no. ab15580, 1:50 PDO)
anti-p-ERK Thr202/Tyr204 (Cell Signaling, cat no. 4370, clone: D13.14.4E, lot: 15, 1:1000)
anti-cl. CASP3 Asp175 (Cell Signaling, cat no. 9661, lot: 47, 1:750 mouse and 1:150 PDO)
anti-RIPK3 (Enzo, cat no. ADI-905-242-100, lot: 03071904, 1:800)
anti-TAK1 (Novus Biologicals, cat no. NBP1-87819, lot: A78535, 1:2250)
anti-phospho TAK1 Ser192 (BIOSS, cat no. bs-5435R, lot: AG07102355, 1:300)
anti-TAB3 (LS Bio, cat no. LS-B4705, lot: 31221, 1:200)

Validation

All commercial available antibodies were validated by the manufacturer.

Eukaryotic cell lines

Policy information about cell lines and Sex and Gender in Research

Cell line source(s)

BxPC-3 (KRASWT, Sanger Cell Lines Project, #COSS906693) - 61-year-old, female PDAC-patient
HPAC (KRASG12D, Sanger Cell Lines Project, #COSS1298136) - 64-year-old, female PDAC-patient
MIA-PACA-2 (KRASG12C, ATTC, #CRL-1420) - 65-year-old, male PDAC-patient
HPDE (generously provided by the laboratory of Dr. Anil K. Rustgi (New York, USA)) - healthy 63-year-old, female
HPDE/GFP and HPDE/KRASG12D (generated by Sebastian Müller, Munich, Germany) - healthy 63-year-old, female
For each cell line, the culture conditions are indicated in Materials and Methods section.

Authentication

Cell lines have been authenticated by ATCC and Sanger Cell Lines Project. Cell lines are futher routinely authenticated in-house by cell morphology.

Mycoplasma contamination

All cell lines are routinely tested negative for mycoplasma contamination.

Commonly misidentified lines
(See ICLAC register)

No commonly misidentified cell lines were used.

Animals and other research organisms

Policy information about studies involving animals; ARRIVE guidelines recommended for reporting animal research, and Sex and Gender in Research

Laboratory animals

LSL-KRASG12D/+, Ptf1a-cre, Tak1fl/fl, Ripk3-/-, Casp8fl/fl and RelAfl/fl strains were interbreed to obtain LSL-KRASG12D/+ Ptf1a-cre (termed KRASG12D), LSL-KRASG12D/+ Tak1fl/fl Ptf1a-Cre (termed KRASG12D TAK1ΔAc), Tak1fl/fl Ptf1a-Cre (termed TAK1ΔAc), LSL-KRASG12D/+ Tak1fl/fl Ripk3-/- Ptf1a-Cre (termed KRASG12D TAK1ΔAc RIPK3-/-), LSL-KRASG12D/+ Tak1fl/fl Casp8fl/fl Ptf1a-Cre (termed KRASG12D TAK1/CASP8ΔAc), LSL-KRASG12D/+ Tak1fl/fl RIPK3-/- Casp8fl/fl Ptf1a-Cre (termed KRASG12D TAK1/CASP8ΔAc RIPK3-/-), LSL-KRASG12D/+ RelAfl/fl Ptf1a-Cre (termed KRASG12D RelaΔAc). Mice were bred on a mixed C57/BL6 - SV129Ola genetic background. In all experiments, littermates carrying the respective loxP-flanked alleles but lacking expression of Cre recombinase were used as wild-type (WT) controls. Age-, gender-, and equal average tumour volume-matched mice were randomly assigned to groups, based on their genotypes and experiments were not blinded. Both male and female mice are included in all groups. Mice age is indicated in the figure legends. A precalculation of the in vivo mouse sample sizes was performed and approved to ensure an optimal balance between the animal welfare guidelines as well as a reasonable sample number for the experiments. Sample sizes and mice age is indicated in the figure legends. All animal experiments were approved by the Federal Ministry for Nature, Environment and Consumers' Protection of the state of North Rhine-Westphalia and were performed in accordance to the respective national, federal and institutional regulations. The maximal permitted tumor size of ≥ 1.5 cm was not exceeded. Mice were housed in individually ventilated cages (IVC) with HEPA-filter from Tecniplast at 22 ± 2 °C, with a humidity of 55 ± 10%, and an air exchange rate of 75 times on a continuous 12 h light-dark cycle from 6am to 6pm.

Wild animals

This study did not involve wild animals.

Reporting on sex | This study did not involve sex research. But Sex-matched mal and female mice of each genotype were generated as littermates for use in experiments in which different genotypes were compared.

Field-collected samples | This study did not involve field-collected sampels.

Ethics oversight | Dr. Lueddes animal protocolls were approved by the local authorities, the Federal Ministry for Nature, Environment and Consumers' Protection of the state of North Rhine-Westphalia as well as the Regierung von Oberbayern.

Note that full information on the approval of the study protocol must also be provided in the manuscript.

Plants

Seed stocks | N/A

Novel plant genotypes | N/A

Authentication | N/A

Flow Cytometry

Plots

Confirm that:

- [x] The axis labels state the marker and fluorochrome used (e.g. CD4-FITC).
- [x] The axis scales are clearly visible. Include numbers along axes only for bottom left plot of group (a 'group' is an analysis of identical markers).
- [x] All plots are contour plots with outliers or pseudocolor plots.
- [x] A numerical value for number of cells or percentage (with statistics) is provided.

Methodology

Sample preparation | FACS on patient-derived tumor spheroids

Patient-derived tumor-spheroids were generated from patient adenocarcinoma pancreatic tissues undergoing surgical resection using a protocol published in 35. Tissue was dissociated using gentleMACS™ Octo Dissociator with Heaters and Human tumor dissociation kit (Miltenyi Biotec, cat no: 130-095-929) following manufacturer's instructions. Total cell populations including cancer cells, fibroblasts and immune cells was used to generate multicellular tumor-spheroids in Corning® 96-well Black/Clear Bottom Low Flange Ultra-Low Attachment Microplate (Corning). Cells were cultured in complete MammoCult™ Human Medium (Stemcell Technologies, cat no: 05620) supplemented with patient serum. After 24h, tumor-spheroids were treated with 5Z-7-Oxozeaenol (25 µM, Sigma, cat no: O9890-1MG) or DMSO (Carl Roth, cat no: 7029.1) as a control overnight. Tumor-spheroids were harvested after treatment and dissociated using accutase. After cell washing using DPBS-/-, antibody cell receptors were blocked using FcR Blocking Reagent (Miltenyi Biotec, cat no: 130-059-901), and CD45+ cells were stained using CD45 Antibody, anti-human APC, REAfinity™ (1:50, Miltenyi Biotec, cat no: 130-110-633, clone: REA747, lot: 5220310224) or with the corresponding REA Control (S) APC antibody (1:50, Miltenyi Biotec, cat no: 130-110-434, clone: REA293, lot: 5220405741), according to manufacturer's instruction. Living cells were selected using Zombie green (BioLegend, cat no: 423111) staining according to manufacturer's instructions. CD45+ cells were enriched by flow cytometry into 384 well cell capture plates (Single Cell Discovery, https://www.scdiscoveries.com) using SH800 cell sorter (Sony) as described35. Each well of a cell capture plate contains a small 50 nl droplet of barcoded primers and 10 µl of mineral oil (Sigma, cat no: M8410). Data were acquired using the Sony SH800 cell sorter software V2.1.5. Sorted plates were briefly centrifuged at 4°C, snap-frozen on dry ice and stored at −80°C until processed.

FACS on patient-derived tumor tissue

Patient adenocarcinoma pancreatic tissues undergoing surgical resection was dissociated using gentleMACS™ Octo Dissociator with Heaters and Human Tumor dissociation kit (Miltenyi Biotec; cat no: 130-095-929) following manufacturer's instructions. The total cell fraction was stained using anti-human APC, REAfinity™ (1:50, Miltenyi Biotec, cat no: 130-110-633, clone: REA747, lot: 5220310224) and Zombie green viability kit (BioLegend, cat no: 423111) according to manufacturer's instruction. Living CD45+ immune cells were separated from other living cell populations using SH800 cell sorter (Sony). Data were acquired using the Sony SH800 cell sorter software V2.1.5. Isolated immune cells were cultured in 96 well plates in complete MammoCult™ Human Medium (Stemcell Technologies, cat no: 05620) supplemented with human proliferation supplement (3.4%, Stemcell Technologies, cat no: 05620), hydrocortisone (0.056%, Stemcell Technologies, cat no: 74142), heparin (0.011%, Stemcell, cat no: 07980), amphotericin B (Merck, cat no: A2942), primocin (InvivoGen, cat no: ant-pm-05) and patient serum). The other cell populations were cultured as tumorspheroids in Corning® 96-well Black/Clear Bottom Low Flange Ultra-Low Attachment Microplate in complete MammoCult™ Human Medium (Stemcell Technologies, cat no: 05620).

After 3 days, tumorspheroids were treated using 5Z-7-Oxozeaenol (25 µM, Sigma, cat no: O9890-1MG) or DMSO (Carl Roth, cat no: 7029.1) as control. Medium was refreshed after 24 h to remove the compounds. After two more days, the conditioned media were used to stimulate immune cells. scRNA-Seq on immune cell population was performed 24h after stimulation. Living cells were sorted into 384 well cell capture plates (Single Cell Discovery, https://www.scdiscoveries.com) using SH800 cell sorter (Sony) as previously described60. Sorted plates were briefly centrifuged at 4°C, snap-frozen on dry ice and stored at −80°C until processed.

Instrument

Cell sorting was performed on Sony SH800 Cell Sorter (Sony, Serial number: 0314067)

Software

Data were acquired and analyzed using Sony SH800 cell sorter software V2.1.5

Cell population abundance

Supplementary Figure 6 (related to Figure 5a-h).
From the dissociated tumorspheroids 76,652 cells were analyzed for DMSO control, and 78,467 cells for 5Z-7-Oxozeaenol-treatment. CD45+ live positive cells were directly sorted in 384 well capture plates.

Supplementary Figure 8 (related to Figure 5i-l).
From the total cell population (437,234 total cells), 206,922 CD45+ living positive cells were isolated with 98.71% of sort efficiency. A post-sort control were performed by an analysis of the post-sort fraction, confirming the purity of the cell fraction.

Gating strategy

Gating strategy is provided in the Supplementray information Figure 6 and 8.

Supplementary Figure 6. Flow cytometry gating strategy for cell sorting (related to Figure 5). a, CD45+ immune cell isolation from tumor patient-derived PDAC tissue by flow cytometry after perturbation studies (Created in BioRender. Schneider, A. (2025) https://BioRender.com/m37a603). After patient-derived tumorspheroid dissociation, total cell population was stained using anti-CD45 antibody coupled with AF647 (APC) and zombie green (ZG, FITC) to detect viable cells. b, The gating was performed on total cell population using FITC-H (zombie green)/FSCA dot plots to remove cell debris and select the viable cells. c, The viable cells were then gated to isolate "singlets" and exclude "doublets" using plot through SSC-W/SSC-H and FSC-W/FSC-H parameters. d, The CD45+ viable immune cells were selected (APC+) using non-stained cells as reference and were sorted in 384 well capture plates for single cell RNA-Seq analysis. Data were analyzed using BD FACSDiva™ Software.

Supplementary Figure 8. Flow cytometry gating strategy for cell sorting (related to Figure 5). a-c, CD45+ immune cell isolation from tumor patient-derived PDAC tissue by flow cytometry (Created in BioRender. Schneider, A. (2025) https://BioRender.com/d09n339). After tissue dissociation, total cell population was stained using anti-CD45 antibody coupled with AF647 (APC) and zombie green (ZG, FITC) to detect viable cells. a, The gating was performed on total cell population using FCS/BSC dot plots to remove cell debris. b, CD45+ and CD45- viable cells were selected (APC+ ZG- and APC- ZG-) using non-stained cells as reference. c, The CD45+ and CD45- viable cells were then gated to isolate "singlets" and exclude "doublets" using plot through FSC-H and FSC-A parameters. d-f, Gating strategy for the sorting of viable immune cells isolated in a-c after perturbation studies (Created in BioRender. Schneider, A. (2025) https://BioRender.com/m37a603). d, After perturbation studies, immune cells were harvested and stained with ZG to detect viable cells e, Viable cells f, The viable cells (ZG-) were then gated to isolate "singlets" and were sorted in 384 well capture plates for single cell RNA-Seq analysis. Data were acquired using Sony SH800 Cell Sorter (Sony).

☒ Tick this box to confirm that a figure exemplifying the gating strategy is provided in the Supplementary Information.
